# Concordance between Phylogeographical and Biogeographical Patterns in the Brazilian Cerrado: Diversification of the Endemic Tree *Dalbergia miscolobium* (Fabaceae)

**DOI:** 10.1371/journal.pone.0082198

**Published:** 2013-12-02

**Authors:** Renan Milagres Lage Novaes, Renata Acácio Ribeiro, José Pires Lemos-Filho, Maria Bernadete Lovato

**Affiliations:** 1 Empresa Brasileira de Pesquisa Agropecuária, Londrina, Brasil; 2 Departamento de Ciências Biológicas, Universidade Federal dos Vales do Jequitinhonha e Mucuri, Diamantina, Brasil; 3 Departamento de Botânica, Instituto de Ciências Biológicas, Universidade Federal de Minas Gerais, Belo Horizonte, Brasil; 4 Departamento de Biologia Geral, Instituto de Ciências Biológicas, Universidade Federal de Minas Gerais, Belo Horizonte, Brasil; University of Lausanne, Switzerland

## Abstract

Few studies have addressed the phylogeography of species of the Cerrado, the largest savanna biome of South America. Here we aimed to investigate the phylogeographical structure of *Dalbergia miscolobium*, a widespread tree from the Cerrado, and to verify its concordance with plant phylogeographical and biogeographical patterns so far described. A total of 287 individuals from 32 populations were analyzed by sequencing the *trnL* intron of the chloroplast DNA and the internal transcribed spacer of the nuclear ribosomal DNA. Analysis of population structure and tests of population expansion were performed and the time of divergence of haplotypes was estimated. Twelve and 27 haplotypes were identified in the cpDNA and nrDNA data, respectively. The star-like network configuration and the mismatch distributions indicated a recent spatial and demographic expansion of the species. Consistent with previous tree phylogeographical studies of Cerrado trees, the cpDNA also suggested a recent expansion towards the southern Cerrado. The diversity of *D. miscolobium* was widespread but high levels of genetic diversity were found in the Central Eastern and in the southern portion of Central Western Cerrado. The combined analysis of cpDNA and nrDNA supported a phylogeographic structure into seven groups. The phylogeographical pattern showed many concordances with biogeographical and phylogeographical studies in the Cerrado, mainly with the Cerrado phytogeographic provinces superimposed to our sampling area. The data reinforced the uniqueness of Northeastern and Southeastern Cerrados and the differentiation between Eastern and Western Central Cerrados. The recent diversification of the species (estimated between the Pliocene and the Pleistocene) and the ‘genealogical concordances’ suggest that a shared and persistent pattern of species diversification might have been present in the Cerrado over time. This is the first time that an extensive ‘genealogical concordance’ between phylogeographic and phytogeographic patterns is shown for the Cerrado biome.

## Introduction

Considerable advances have been made in disentangling biogeographical patterns across the globe, but the diversification of many biodiversity rich areas of the world remain poorly studied [1]. One such region is the Cerrado biome, a savanna vegetation that, together with the Seasonally Dry Tropical Forests (SDTF), covers a huge diagonal seasonally dry area in the interior of Eastern Tropical South America (ETSA) [1]–[3]. The Cerrado is typically found in dystrophic soils, often latosols, under a climate characterized by a marked dry season, whereas the SDTFs typically occur in areas with more fertile soils and longer dry seasons [2]–[4]. The Cerrado is the most diverse savanna in the world, particularly in terms of the number of plant species present [5], and it has high levels of endemisms and species adapted to stressed environments [3], [5], [6]. Its almost 1.8 million km^2^ of original area has been reduced by more than 50% in the last years [7], making the Cerrado one of the main global hotspots for biodiversity conservation [5]. Despite this relevance, many details about the historical biogeography of the Cerrado biome are unresolved [4], [8].

**Figure 1:**
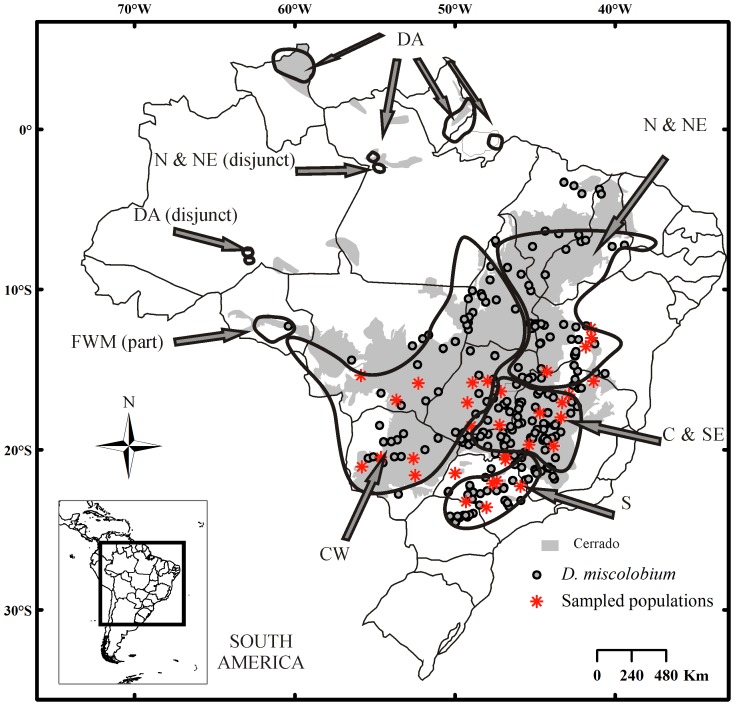
Geographic distribution of *Dalbergia miscolobium* (dots) across the Brazilian Cerrado biome (grey) in Eastern Tropical South America. Each dot represents a record of *D. miscolobium* and each red asterisk a sampled population. The geographic distribution of *D. miscolobium* was estimated through field trips, herbarium records and databases of floristic checklists. The six phytogeographical provinces proposed by Ratter et al. [11] have been delimited: ‘S’, Southern, ‘C & SE’, Central and southeastern, ‘N & NE’, North and northeastern, ‘CW’, Central-western, ‘FWM’, Far western mesotrophic sites and ‘DA’, Disjunct Amazonian. The codes of the provinces are the same of the original publication.

The Cerrado has a highly heterogeneous biota and many studies have tried to subdivide it into biogeographic provinces (see [4], [8] for reviews). Three broad phytogeographical studies have found similar patterns [9]–[11]. In studies analyzing 145 Cerrado areas, Castro [9] and Castro and Martins [10] recognized three main phytogeographic groups, a Southern Cerrados group (named ‘São Paulo’), a Central Cerrados group (named ‘Planalto Central’) and the Northeastern Cerrados. Largely concordant with it, Ratter *et al.* [11], based on the floristic similarity of a broader sampling effort involving 376 Cerrado areas, identified six phytogeographic provinces (see names and delimitation of them in Figure 1). Their results were largely concordant and they suggested that climate and soil, as well as past climatic events, might be among the main factors responsible for the patterns found. With regard to the impact of past climates on the Cerrado biota, many controversies exist, and expansion [12], stability [13], and reduction [14], [15] of its extension during the Last Glacial Maximum (LGM) have been proposed. Phylogeographical approaches would help to evaluate the persistence of these patterns at the intra-specific level and to help unravel the causes for them.

Phylogeographical studies have proved to be valuable independent sources of evidence in comprehending biogeographical patterns and in reconstructing past vegetation dynamics [16]. Concordances between phylogeographical and biogeographical patterns and paleodata have been found in numerous instances (e. g. [17], [18]), thus helping understand the evolution and shared history of many species. Nevertheless, only a few studies have addressed the phylogeography of species from the Cerrado [1], [8] and phylogeographical studies that have focused on Cerrado trees are scarce [19]–[22]. Essentially, these studies found phylogeographic structuring and high levels of differentiation among populations in the respective species analyzed. Three of them observed major groups of populations subdivided longitudinally [20]–[22], and three [19], [21], [22] observed also patterns of recent range expansion of northern resources towards the southern Cerrado. These expansions have been hypothesized to have occurred after the return of wetter and warmer climatic conditions after the last glacial period. A better understanding of the evolutionary patterns of the Cerrado could be achieved with more phylogeographical studies of species with distinct ecological requirements together with wider samplings across the biome, including the peripheral regions. In the present study, we focused on a widespread tree species adapted to the coldest regions of the biome, and encompassing a large portion of the biome, including the southwest portion.

The species chosen for this study, *Dalbergia miscolobium* Benth. (Papilionoideae, Fabaceae), is endemic and widespread in the Cerrado (Figure 1). It is among the 121 most representative tree species of the biome and is the 9^th^ and 12^th^ most common tree species in the southeastern and the southern Cerrado, respectively [23]. Unlike other Cerrado trees subjects of phylogeographical studies so far, *D. miscolobium* seems to be more tolerant to the cold. It is common in the coldest (subject to eventual frosts during winter) and southernmost portions of the Cerrado, and is widely found at higher altitudes (>1,000 m) in the biome [24]. In the work of Bridgewater *et al.* [23], *D. miscolobium* was found in 82% of the 22 floristic surveys conducted in the southern Cerrado and in 75% of the 73 sites studied in the south-eastern Cerrado. On the other hand, the species was encountered in only 37% and 28% of the north-eastern and central-western Cerrado provinces, respectively. It is a tree pollinated by bees and its seeds are wind-dispersed [25]. It is an important timber species, and is a rapid colonizer of suitable environments.

Here, we undertook an extensive sampling of *D. miscolobium* populations encompassing a large portion of the Cerrado biome in order to investigate the species' phylogeographical structure and to verify its congruence with phylogeographical and biogeographical patterns reported to the Cerrado biome so far. More specifically, we aimed to answer the following questions: 1) is the phylogeographical structure of this wide range species congruent with the Cerrado biogeographical provinces proposed by Ratter *et al.* [11]? Considering that the historical casual factors that contributed to determine the phytogeographical provinces of Cerrado may also have contributed to the phylogeographical structure of *D miscolobium* we predicted some congruency between them; 2) in what extension is the phylogeographical structure of *D. miscolobium* similar to other Cerrado tree species studied so far? Based on previous studies [20]–[22] we predicted that the southern populations present lower genetic diversity than populations from central Cerrado, which in turn would present high diversity levels, consistent with a recent colonization of southernmost populations from more stable northernmost areas.

## Materials and Methods

### Ethics Statement

*Dalbergia miscolobium* is not included in the Brazilian Official List of Threatened Plants. The collection of samples of leaves in conservation units was realized with the legal authorization of the ‘Instituto Brasileiro do Meio Ambiente e dos Recursos Naturais Renováveis’ (IBAMA; populations DFE, MUC and PER - licenses 192/2003, 110/2005, 26.627.1) and of the ‘Instituto Estadual de Florestas – MG’ (population RPE – license 018/05). The collection of the samples in localities out of conservation units had authorization for collection and transport of IBAMA (licenses 099/2004 and 016/2006, 14035-2, 293770) and of the owners of the lands. Also we had authorization for the access to sampling of component of genetic patrimony of the Brazilian ‘Conselho de Gestão do Patrimônio Genético’ (CGEN/MMA) number 03/2004.

### Population sampling and DNA amplification

The geographic distribution of *D. miscolobium* was estimated through field trips, databases of floristic checklists (personal communications by José Felipe Ribeiro and Ary T. Oliveira-Filho) and herbarium records (Figure 1). Expeditions were carried out from 2004 to 2010 to sample *D. miscolobium* across the Brazilian Cerrado. Leaf samples were collected from 287 adult trees in 32 populations covering most of its geographic distribution range, encompassing seven Brazilian states (Table 1 and Figure 1). Our sampling covered all the four Cerrado phytogeographic provinces proposed by Ratter *et al.* [11] in which the species is found: Southern (S), Central and southeastern (C & SE), North and northeastern (N & NE) and Central-western (CW) (Figure 1). We sampled a lower number of populations in the N & NE province, where the species is scarcely found (see Introduction). Three to 14 individuals were sampled in each population. Populations were sampled in natural reserves and remnant fragments of the Cerrado (Table 1). Genomic DNA was isolated by a modified cetyl trimethylammonium bromide (CTAB) extraction method [26] and quantification was carried out by visual inspection of agarose gels.

**Table 1:** Locations of *Dalbergia miscolobium* populations in the Brazilian Cerrado biome, genetic diversity indices and the distribution of the chloroplast DNA (cpDNA) and nuclear ribosomal DNA (nrDNA) haplotypes.

			cpDNA data			nrDNA data		
*Locality, State* a *(population code)*	*Latitude (S) - Longitude (W)*	*Sample size* b	*Haplotype diversity*	*Nucleotide diversity (X10^−2^)*	*Haplotypes*	*Haplotype diversity*	*Nucleotide diversity (X10^−2^)*	*Haplotypes*
Água Clara, MS (AGU)	20°32′36″– 52°34′26″	14	0.473	0.083	C1, C2, C3	0.712	0.155	N1, N2, N3, N4, N5
Bataguassu, MS (BAT)	21°36′05″– 52°28′19″	07	0.000	0.000	C1	0.495	0.080	N1, N2
Nioque, MS (NIO)	21°04′04″– 55°47′38″	11	0.327	0.054	C1, C3	0.710	0.175	N1, N2, N3, N4
Anhanduí, MS (NHA)	20°28′15″– 54°37′26″	06	0.000	0.000	C4	0.712	0.181	N1, N2, N3, N4
Alto das Garças, MT (GAR)	16°53′58″– 53°38′59″	10	0.000	0.000	C5	0.747	0.171	N1, N3, N4, N6
Barra das Garças, MT (BGA)	15°51′19″– 52°16′09″	10	0.000	0.000	C6	0.000	0.000	N4
Salgadeira, MT (CGU)	15°22′02″– 55°50′25″	10	0.000	0.000	C1	0.468	0.080	N3, N4, N6
Professor Jamil, GO (PJA)	17°04′03″– 49°13′24″	11	0.000	0.000	C6	0.000	0.000	N4
Pirenópolis, GO (PNO)	15°48′44″– 48°53′28″	10 (7)	0.000	0.000	C6	0.200	0.032	N4, N6
Brasília, DF (DFE)	15°43′39″– 47°56′44″	10	0.000	0.000	C1	0.440	0.071	N3, N7
Avanhandava, SP (AVA)	21°28′45″– 49°58′13″	06	0.000	0.000	C2	0.621	0.115	N1, N2, N6
Analândia, SP (ANA)	22°07′40″– 47°38′48″	10	0.000	0.000	C7	0.647	0.210	N8, N9, N10, N11
Emas, SP (EMA)	21°56′01″– 47°22′23″	10	0.000	0.000	C7	0.614	0.200	N8, N9, N10, N12, N13
Itapetininga, SP (IPE)	23°35′49″– 48°01′42″	10	0.000	0.000	C7	0.100	0.016	N9, N12
Piraju, SP (PIR)	23°15′12″– 49°18′16″	10	0.000	0.000	C7	0.358	0.116	N9, N10, N12
Bom Despacho, MG (BDE)	19°44′00″– 45°15′00″	07	0.571	0.094	C1, C8	0.485	0.078	N3, N7
Coromandel, MG (COR)	18°29′03″–47°12′10″	10	0.533	0.088	C7, C9	0.574	0.152	N3, N7, N14, N15, N16
Divisa Alegre, MG (DAL)	15°43′32″– 41°20′42″	10 (9)	0.000	0.000	C7	0.835	0.236	N3, N7, N14, N17, N18, N19
Delfinopólis, MG (DEL)	20°20′45″– 46°50′15″	03	0.667	0.110	C1, C10	0.333	0.054	N3, N7
Grão Mogol, MG (GMO)	16°34′00″– 42°53′00″	06	0.533	0.088	C1, C7	0.682	0.132	N3, N14, N17, N19
Itacambira, MG (ICA)	15°06′00″– 44°06′00″	06 (5)	0.000	0.000	C7	0.750	0.156	N3, N17, N18
Pouso Alegre, MG (PAL)	22°16′26″– 45°53′27″	10	0.000	0.000	C7	0.774	0.353	N9, N10, N12, N20, N21, N22, N23
Januária - Peruaçu, MG (PER)	15°07′20″– 44°14′53″	10	0.000	0.000	C1	0.495	0.080	N3, N17
São Gonçalo do Rio Preto, MG (RPE)	18°00′00″– 43°23′00″	10	0.200	0.033	C1, C7	0.673	0.154	N3, N7, N14, N17, N18
Santa Luzia, MG (SAL)	19°46′00″– 43°51′00″	06	0.533	0.088	C1, C8	0.409	0.066	N3, N7
São João Batista da Glória, MG (SJO)	20°37′21″– 46°31′13″	03	0.000	0.000	C5	0.600	0.097	N3, N7
Tupaciguara, MG (TUP)	18°31′32″– 48°59′29″	12 (11)	0.000	0.000	C6	0.000	0.000	N4
Unaí, MG (UNA)	16°22′14″– 47°07′21″	10 (8)	0.467	0.154	C7, C11	0.791	0.199	N3, N7, N17, N18
Várzea da Palma, MG (VPA)	17°42′37″– 44°41′24″	10	0.533	0.088	C1, C7	0.739	0.200	N3, N14, N17, N18
Mucugê, BA (MUC)	13°03′13″– 41°28′30″	10	0.000	0.000	C12	0.667	0.224	N3, N7, N24, N25, N26
Palmeiras, BA (PRA)	12°26′36″– 41°29′49″	10 (9)	0.000	0.000	C12	0.933	0.345	N3, N7, N24, N25, N27
Rio de Contas, BA (RCO)	13°35′21″– 41°49′07″	09	0.000	0.000	C12	0.837	0.272	N3, N7, N14, N24, N25, N26

a Abbreviation of Brazilian States: MS, Mato Grosso do Sul; MT, Mato Grosso; GO, Goiás; DF, Distrito Federal; SP, São Paulo; MG, Minas Gerais; BA, Bahia.

b In parentheses sample size analyzed through nrDNA when this was different from that of cpDNA.

The following regions were chosen for DNA sequencing: the *trnL* intron of the chloroplast DNA (cpDNA) and the internal transcribed spacer (ITS) region of the 18S–26S nuclear ribosomal DNA (nrDNA) genes (which includes the ITS1 and ITS2 intergenic spacers and the 5.8S gene). The *trnL* intron and ITS region were amplified and sequenced using primers as described by Taberlet *et al.* [27] and Delgado-Salinas *et al.* [28], respectively. The cpDNA region was chosen, because it presented specific amplification and the highest variability among 15 non-coding cpDNA regions evaluated (information is available on request). Special care was taken with regard to the specificities of the ITS region and the main guidelines proposed by Feliner and Rosselló [29] were followed in the amplification and analysis of this region. The amplification of both regions was performed in 25 µl total volume containing 10–20 ng of DNA, 1X PCR buffer with 2.0 mM MgCl_2_, 200 µM of each dNTP, 0.2 ng bovine serum albumin (BSA), 0.5 µM of each primer, 1 U Taq DNA polymerase (Phoneutria, Belo Horizonte, Brazil), and autoclaved deionized water. The nrDNA region reaction mixture also contained 2% dimethyl sulfoxide (DMSO) and 1 M Betaine (N3-trimethyglycine). Thermal conditions of the reaction were as follows: initial denaturation at 94°C for 2 min, followed by 35 cycles at 94°C for 1 min, 56°C (cpDNA) or 60°C (nrDNA) for 1 min, and 72°C for 1 min, and a final elongation at 72°C for 7 min. Each PCR product was then double-strand sequenced using the DYEnamic ET dye terminator sequencing Kit (GE Healthcare, UK). Sequencing reactions were analyzed on a MegaBACE 1000 automated sequencer (GE Healthcare) following the manufacturer's instructions.

### Sequence analysis

Sequences were assembled using the package PHRED/PHRAP/CONSED to generate consensus sequences. The cpDNA and nrDNA sequences were deposited in GenBank under accession numbers JQ612719 to JQ612730 and JQ582850 to JQ582876, respectively. The consensus sequences were aligned using CLUSTAL-W [30] implemented in the MEGA 5 program [31], followed by careful manual adjustment. The ends of the sequences were pruned to eliminate fragments that could not be obtained for all individuals and to preserve only high confidence bases.

For nrDNA sequences, all variable sites were carefully checked in the original electropherograms and, when heterozygous nucleotide positions identified by double peaks were present, samples were re-sequenced independently in order to confirm the genotype. To reconstruct the nrDNA haplotypes, we used PHASE 2.1 software [32], which uses a Bayesian statistical method to infer haplotypes of individuals that have more than one polymorphic site based on genotypic data. It was run under default conditions, allowing for multiallelic loci (-d option) and for 10,000 iterations. In the first run, one multiallelic site was shown to be highly homoplasious in our data and, based on this, we removed this site prior to further analysis. Only haplotypes recovered with >0.90 posterior probability were used in subsequent analyses, a precaution adopted by other studies that have used PHASE [33]. To perform subsequent genetic analyses, the output of PHASE was transformed through the pipeline described by Machado *et al.* [34].

### Population genetic and phylogeographical analyses

Genetic diversity indices were estimated in ARLEQUIN 3.5 [35] and in DNAsp 5.10 [36]. The hypothesis of population expansion was tested by pairwise mismatch distributions [37] and the neutrality tests Tajima's *D* [38] and Fu's *F_S_* [39]. These analyses were performed with the ARLEQUIN and DNAsp programs. Allelic richness was computed in Contrib [40] and index of differentiation among populations was estimated by D [41] in SMOGD [42]. Both the indices and the tests were performed for the whole species as well as for the phylogeographic groups of populations delimited in the analyses (see Results and Table 2 for details about their composition). Analyses of molecular variance (AMOVA; [43]) using pairwise differences were conducted in ARLEQUIN to assess population genetic structure in *D. miscolobium* based on cpDNA and nrDNA data.

**Table 2:** Genetic diversity indices for the phylogeographic groups of *Dalbergia miscolobium* based on chloroplast DNA (cpDNA) and nuclear ribosomal DNA (nrDNA) data.

Phylogeographic groups^a^	Data	Sample size	Total haplotypes	Shared haplotypes	Exclusive haplotypes	Haplotype diversity (SD)	Nucleotide diversity (SD) (X10^−2^)	Allelic richness^c^
**All populations**	cpDNA	287	12	-	-	0.794 (0.013)	0.289 (0.190)	6.542
	nrDNA	251^b^	27	-	-	0.893 (0.006)	0.510 (0.290)	8.905
**Central Eastern 1 (CE1)**	cpDNA	62	3	C1,C7	C11	0.516 (0.042)	0.095 (0.086)	1.696
	nrDNA	49	6	N3,N7,N14	N17,N18,N19	0.758 (0.024)	0.185 (0.134)	3.954
**Central Eastern 2 (CE2)**	cpDNA	39	6	C1,C5,C7	C8,C9,C10	0.739 (0.056)	0.193 (0.141)	4.599
	nrDNA	35	5	N3,N7,N14	N15,N16	0.521 (0.047)	0.104 (0.091)	2.101
**Central Western 1 (CW1)**	cpDNA	20	2	C1,C5	-	0.526 (0.036)	0.087 (0.084)	1.000
	nrDNA	17	4	N1,N3,N4,N6	-	0.658 (0.064)	0.144 (0.114)	2.874
**Central Western 2 (CW2)**	cpDNA	44	4	C1	C2,C3,C4	0.599 (0.066)	0.315 (0.203)	2.826
	nrDNA	44	6	N1,N3,N4,N6	N2,N5	0.705 (0.021)	0.164 (0.123)	2.963
**Central Cerrados (CC)**	cpDNA	43	1	-	C6	0.00	0.00	0.00
	nrDNA	37	2	N4,N6	-	0.027 (0.026)	0.044 (0.016)	0.230
**Southern Cerrados (SC)**	cpDNA	50	1	C7	-	0.00	0.00	0.00
	nrDNA	49	10	-	N8,N9,N10,N11, N12,N13,N20, N21,N22,N23	0.596 (0.049)	0.235 (0.159)	3.587
**Northeastern Cerrados (NC)**	cpDNA	29	1	-	C12	0.00	0.00	0.00
	nrDNA	20	7	N3,N7,N14	N24,N25,N26, N27	0.815 (0.034)	0.297 (0.193)	5.023
**Central Eastern (CE1+CE2)**	cpDNA	101	7	C1,C5,C7	C8,C9,C10,C11	0.675 (0.029)	0.146 (0.114)	4.532
	nrDNA	84	8	N3,N7,N14	N15,N16,N17, N18,N19	0.751 (0.017)	0.193 (0.137)	5.026
**Central Western (CW1+CW2)**	cpDNA	64	5	C1,C5	C2,C3,C4	0.638 (0.055)	0.271 (0.179)	3.817
	nrDNA	61	6	N3,N4,N6	N1,N2,N5	0.780 (0.012)	0.205 (0.144)	4.291

a The phylogeographic groups are composed by following populations: **CE1** = DAL, GMO, ICA, PER, RPE,UNA,VPA; **CE2** = BDE, COR, DEL, SAL, SJO, DFE; **CW1** = GAR, CGU; **CW**2 = AGU, BAT, NIO, NHA, AVA; **CC** = BGA, PNO, PJO, TUP; **SC** = ANA, EMA, IPE, PIR, PAL; **NC** = MUC, PRA, RCO. See also the Figure 3D.

b Twenty-seven individuals were excluded in PHASE analysis due to unresolved genotypes.

c Allelic richness after rarefaction to 29 and 20 for cpDNA and nrDNA, respectively.

The phylogenetic relationships among haplotypes were inferred using the Median-Joining (MJ) network method [44] and Statistical Parsimony [45] as implemented in NETWORK 4.6 (fluxus-engineering.com) and TCS 1.21 [46], respectively. Insertion/deletions (indels) were considered as fifth character states and were coded as single mutations, regardless of their size.

The phylogeographic structure of populations was evaluated through a Bayesian Analysis of genetic Population Structure in BAPS 5.3 [47]. Despite the availability of some other software for Bayesian spatial analyses [48], we opted to use BAPS because of its efficiency in unveiling population structures [49] and its extensive usage for definition of clustering of genetic variation using geographical information (e.g. [50]–[53]). The analysis was performed at the group level, considering each population as a group, and using their respective geographical coordinates. The BAPS analysis was performed for the cpDNA and nrDNA data both separately and concatenated. The best number of clusters (k) was determined after 15 runs under a maximum of 20 clusters. We also made some analysis with prior numbers of clusters based on the number of Cerrado phytogeographic provinces superimposed to our sampled area (k = 4 equivalent to Ratter *et al.*'s consensus analysis and k = 5 to Ratter *et al*.'s Twinspan analysis; see Results for details). The location of potential geographic barriers was explored through the Monmonier's algorithm in BARRIER 2.2 [54]. As genetic data inputs, we used Nei unbiased distance matrices [55] generated through GenALEx 6.5 [56]. We used the matrices of cpDNA and nrDNA together to compute one to ten barriers. To confirm the phylogeographical structure and to test the hypothesis of concordance between phylogeography and biogeography, hierarchical AMOVAs [43] were conducted in ARLEQUIN, following Brante *et al.* [57]. The AMOVAs were run with the same clusters identified by BAPS with cpDNA, nrDNA and concatenated data, as well as with the population clusters established by BAPS in the analysis with prior of 4 and 5 clusters (see above). The fixation indices F_CT_, F_SC_ and F_ST_ were computed considering the pairwise distances between haplotypes. The level of significance was computed by 1000 nonparametric permutations.

The divergence times among the cpDNA and nrDNA haplotypes identified in *D. miscolobium* were estimated through a Bayesian coalescent approach implemented in BEAST 1.6 [58]. We assumed a Bayesian skyline plot tree prior with two groups and a piecewise-linear model. Independent runs were carried out for the cpDNA and nrDNA datasets, both under a HKY substitution model and a strict clock, and each composed of three runs in a total of 2.2×10^8^ generations. Substitution rates for the *trnL-trnF* and ITS regions of *Inga* species, estimated to be 1.3×10^−9^ and 2.34×10^−9^ substitutions per site per year, respectively [59], were used to estimate divergence times. The genus *Inga* shares many features with *Dalbergia*. In particular, both species belong to the Leguminosae family, are composed of trees, and occur in the neotropics. Thus, the estimated rates for sequence evolution of *Inga* are suitable for use in our study.

## Results

### Genetic diversity

Consensus sequences were obtained from 287 and 278 individuals of *D. miscolobium* for the cpDNA and nrDNA data, respectively. For cpDNA, the alignment produced a total of 606 bp with 13 detected polymorphic sites, which included 11 base substitutions and two indels (Table S1). Twelve cpDNA haplotypes were shared across *D. miscolobium* populations. For the nrDNA region, in a total alignment of 618 bp, we found 15 polymorphic sites (all represented by base substitutions), resulting in 27 haplotypes (Table S2). After the PHASE analysis, 27 sampled individuals (54 allele copies) were excluded from the nrDNA analysis due to unresolved genotypes (p<0.90), thus leaving 251 individuals in the analysis totaling 502 allele copies. Because of the nature of nrDNA region, some heterozygotes we observed could be in fact paralogous copies that escaped concerted evolution [29], and because of that we did not perform any analysis concerning the heterozygosity of sampled individuals.

The molecular diversity indices and haplotypes distributions for each *D. miscolobium* population are described in Table 1. The majority of the populations had no genetic variability in the cpDNA data (22 populations of the 32 analyzed), whereas only three populations (BGA, PJA, and TUP) were monomorphic in the nrDNA data (Table 1). For the cpDNA and nrDNA data, the total haplotype diversity was 0.794 and 0.893, and the total nucleotide diversity was equal to 0.00289 and 0.00510, respectively (Table 2). The *F_ST_* values were estimated in 0.952 and 0.740 for cpDNA and nrDNA respectively, showing high differentiation among populations [60], which were corroborated by the Jost's D values of 0.799 and 0.805 for cpDNA and nrDNA data, respectively.

### Phylogeographical structure

**Figure 2:**
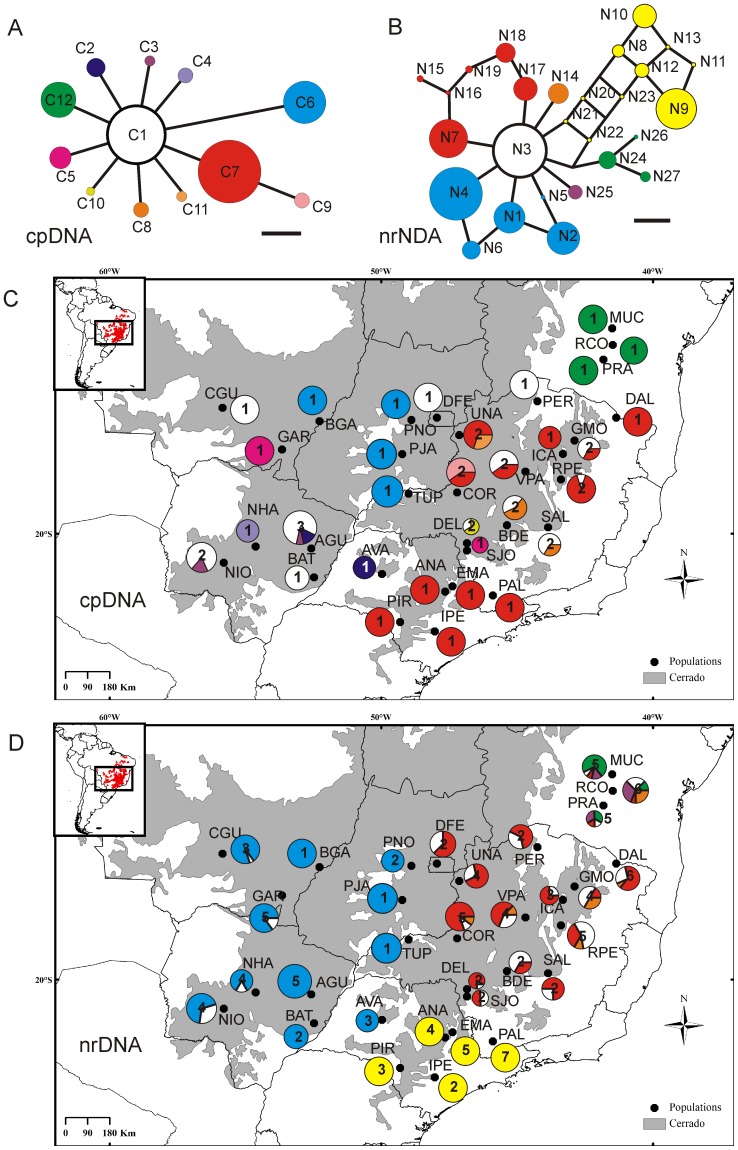
Median-joining networks depicting the relationships among haplotypes of *Dalbergia miscolobium* based on cpDNA (A) and nrDNA (B) data. Geographic distribution of the cpDNA (C) and nrDNA (D) haplotypes across sampled populations. Population and haplotype codes correspond to those in Table 1 and Table 2. The network colours are equivalent to those at the maps for the respective genomes. The full circles are proportional to the number of individuals. The numbers inside the circles correspond to number of haplotypes found in that population.

The phylogenetic relationships among haplotypes inferred through both network methods had very similar topologies; therefore, only the MJ networks are shown (Figure 2A, B). For both cpDNA and nrDNA data, the networks exhibited a central haplotype that was common and the most widespread, and from which many other haplotypes diverged in a star-like fashion. For cpDNA data, all haplotypes (except C9) were directly linked to central haplotype (C1), and only C6 was separated from it by more than one mutation event (three base substitutions; Figure 2A and Table S1). For nrDNA, the pattern was similar but the number and level of divergence of haplotypes were greater (Figure 2B and Table S2). Many reticulations connected the groups of haplotypes that derived from the central haplotype (Figure 2B), indicating equally probable evolutionary pathways. These reticulations at the nrDNA level have been observed in other plant species (e.g.: [61], [62]) and we believe that it does not affect our main conclusions.

Similar phylogeographical structure was observed in the cpDNA and nrDNA data (Figure 2C, D). In both data sets the central haplotype was very common and was widespread in the central, western, and southeastern regions of the Cerrado. The ‘peripheral haplotypes’ in the nrDNA network were, in general, more restricted to certain geographic regions and most of them also less frequent. Furthermore, the haplotypes were not randomly distributed, and a clear structure could be observed (Figure 2).

**Figure 3:**
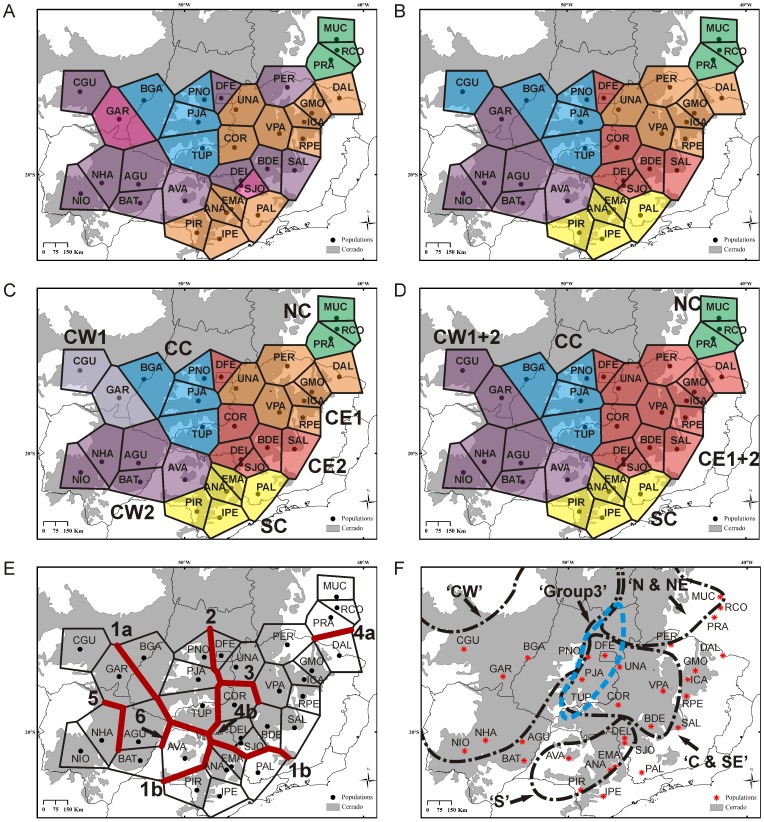
Results from population structure analyses of *Dalbegia miscolobium* populations (A to E) and the phytogeographical provinces proposed by Ratter *et al.* [11] that are superimposed to our sampling area (F). In each panel is represented a result of a different analysis: A) Bayesian Analysis of Population Structure (BAPS) with cpDNA only; B) BAPS with nrDNA only; C) BAPS with cpDNA and nrDNA concatenated; D) BAPS with cpDNA and nrDNA concatenated with a prior of k = 5; E) Analysis with the Monmonier's algorithm implemented in Barrier 2.2. In panels A to E, the polygons are the result of Voronoi tessellation and each one is correspondent to a population whose code is the same as in Table 1. Polygons with the same color belong to the same BAPS cluster. In panel E, the red lines correspond to the consensus barriers among the cpDNA and nrDNA distance matrices. The barriers are numbered in order of their appearance which is related to their probability of existence. The larger abbreviations in panels C and D are for the phylogeographic groups as detailed in Results and Table 2. In panel F, the black traces are for the phytogeographical provinces of Ratter *et al.* [11] and the blue trace is correspondent to Group 3 from their Twinspan analysis. The codes of the provinces are the same of the original publication. For names of provinces see Figure 1 and for further details about them, refer to the text.

The Bayesian Analysis of Population Structure (BAPS) pointed to the most probable existence of five and six clusters of populations for the cpDNA and the nrDNA datasets, respectively (Figure 3A, B). With the data concatenated, the analysis suggested seven clusters (Figure 3C). Two clusters, ‘Northeastern Cerrados’ and the ‘Central Cerrados’, remained the same in the three types of datasets used (cpDNA, nrDNA and concatenated; Figure 3A–C). The divisions between ‘Central-Eastern’ and ‘Central-Western’ as well as between ‘Central-Eastern’ and ‘Southern Cerrados’ were weaker for the cpDNA data, showing signs of previous connections and relationships between those regions. The nrDNA and the concatenated results were very similar to each other (Figure 3B, C). As the concatenated clusterization takes into account both genomes and as there was a considerably concordance among the analyses for the three datasets (cpDNA, nrDNA and concatenated), we selected the concatenated results (Figure 3C) as the best representation of *D. miscolobium* phylogeographical structure. The AMOVA showed that the most of genetic variation was distributed among phylogeographical groups for both cpDNA (83.4% and 75.6% for five and seven clusters, respectively) and nrDNA (71.7% and 71.2% for six and seven clusters, respectively) (Table 3), confirming the consistency of the clusters determined by BAPS.

**Table 3:** Analyses of molecular variance (AMOVA) of cpDNA and nrDNA data among clusters identified by Bayesian Analysis of Population Structure (BAPS) with cpDNA, nrDNA and concatenated data, as well as with the population clusters established by BAPS in the analysis with prior of 4 and 5 clusters.

BAPS clusters	Source of variation	Variation %	*F*-statistics^a^
**cpDNA data**			
5 clusters	Among groups	83.4	*F_CT_* = 0.83
	Among populations within groups	12.2	*F_SC_* = 0.73
	Within populations	4.4	*F_ST_* = 0.95
7 clusters	Among groups	75.6	*F*_CT_ = 0.75
	Among populations within groups	19.3	*F_SC_* = 0.78
	Within populations	5.1	*F_ST_* = 0.94
4 clusters a priori	Among groups	45.8	*F_CT_* = 0.45
	Among populations within groups	49.2	*F_SC_* = 0.90
	Within populations	5.0	*F_ST_* = 0.95
5 clusters a priori	Among groups	76.2	*F_CT_* = 0.76
	Among populations within groups	19.0	*F_SC_* = 0.79
	Within populations	4.8	*F_ST_* = 0.95
**nrDNA data**			
6 clusters	Among groups	71.7	*F_CT_* = 0.71
	Among populations within groups	5.1	*F_SC_* = 0.17
	Within populations	23.2	*F_ST_* = 0.76
7 clusters	Among groups	71.2	*F_CT_* = 0.71
	Among populations within groups	5.2	*F_SC_* = 0.18
	Within populations	23.6	*F_ST_* = 0.76
4 clusters a priori	Among groups	67.4	*F_CT_* = 0.67
	Among populations within groups	11.2	*F_SC_* = 0.34
	Within populations	21.4	*F_ST_* = 0.78
5 clusters a priori	Among groups	68.2	*F_CT_* = 0.68
	Among populations within groups	9.5	*F_SC_* = 0.29
	Within populations	22.3	*F_ST_* = 0.77

a *P*-value<0.0001 for all analyses.

See methods and results for more details about the clusters.

We named each one of the seven clusters using as a basis the biogeographic units described by Ratter *et al.* [11], as they have been used as a reference to some subsequent biogeographic studies (e. g. [63]), and we will use that nomenclature from now on to refer to them (Table 2 and Figure 3C). The groups Central Cerrados (CC), Southern Cerrados (SC) and Northeastern Cerrados (NC) are the first ones to be separated from the rest of the populations in the BAPS analyses with lower values of k (k<7), what highlights their consistency as distinct phylogeographic units. Moreover each one has very peculiar characteristics with regard to levels and patterns of genetic diversity in cpDNA and nrDNA, relationships with the other provinces and haplotypes composition and uniqueness (Figures 2 and 3 and Table 2). The other four groups, CW1, CW2, CE1 and CE2, on the other hand, appear to be more connected to each other, as they share the most common and widespread haplotypes of both cpDNA and nrDNA genomes (Figure 2A, B). These groups do not have a genetic uniformity and they do have some neighboring populations with completely different genetic compositions. Probably because of that, in BAPS analyses, they are not separated with cpDNA data, and the subdivisions between CW1 and CW2 groups as well as between CE1 and CE2 groups do not appear in BAPS analysis with concatenated data with lower k values (k = 5 and k = 4).

In order to test for the fitness of our data to the Cerrado phytogeographical provinces proposed by Ratter *et al.* [11] we also ran BAPS using as priors four and five clusters for the concatenated data. By doing this we aimed to verify if our seven groups could be partitioned into Ratter *et al*'s phytogeographical Cerrado provinces. Those priors correspond to the number of Cerrado's provinces that are superimposed to our sampling area in two ways: Ratter et al's Consensus results (k = 4; the four provinces ‘CW’, ‘S’, ‘C & SE’ and ‘N & NE’) and Twinspan results (k = 5; province ‘C & SE’ is subdivided; Figure 3F). Compared to the partition without priors (seven groups; Figure 3C), the prior of four clusters (k = 4) resulted in the merging of the groups NC, CE1 and CE2 into one and the groups CW1 and CW2 into another (Figure S1). With the prior of five clusters (k = 5), group NC is detached from CE1+CE2 (Figure 3D). Both configurations, but mainly the five clusters partition, considerably match the provinces of Ratter *et al.* [11] as analyzed by the Twinspan method (Figures 3D, F and S1). The AMOVAs with the five clusters partition showed high F_CT_ values for cpDNA (F_CT_ = 0.76) and nrDNA (F_CT_ = 0.68), which was higher than with the four clusters division (F_CT_ = 0.45 and 0.67, respectively; Table 3), reinforcing greater concordance of the phylogeographical groups with the provinces proposed by Ratter *et al.* [11], analyzed by the Twinspan method.

To further investigate the support of those subdivisions we explored the potential place of genetics barriers through the Monmonier's algorithm implemented in BARRIER. We added barriers from one to ten, using multiple matrices (cpDNA and nrDNA), and we show only the consensus barriers among the matrices (Figure 3E). With the adding of 10 barriers, six are consensual among cpDNA and nrDNA and the subdivisions determined by them are almost the same as those observed in BAPS concatenated seven clusters. Altogether, the four most probable barriers are placed almost exactly at the limits among BAPS five clusters (Barriers 1 to 4 in Figure 3E).

Exclusive haplotypes could be found in six of the seven groups (Table 2) showing a widespread distribution of diversity. But only two centres of genetic diversity were common for cpDNA and nrDNA data. The first centre was composed by the CE1 and CE2 groups, comprised mainly of populations from the state of Minas Gerais, and the second was the CW2 group. These centers have many exclusive haplotypes in addition to the central one, higher level of intrapopulation variation and high levels of genetic diversity (Table 2, Figure 2C, D). Two other groups, SC and NC were completely monomorphic for the cpDNA but medium (SC) to highly (NC) diverse for nrDNA (Table 2).

### Population expansion and time of divergence

Population expansion, both demographic and spatial, of the species was corroborated by the mismatch distributions for the cpDNA and nrDNA data, where the observed values did not differ from the expected values under a sudden-expansion model. For the phylogeographic groups, only the CW2 and CE1 for nrDNA and CW1 for cpDNA did not exhibit population expansion pattern by mismatch distributions, as well as the groups of CE1+CE2 and CW+CW2 for both cpDNA and nrDNA (Table 4). The neutrality tests (Tajima's *D* and Fu's *F_S_*), on the other hand, did not have significant values for all populations together or for any of the seven groups (data not shown), and thus do not support any population expansions. The neutrality tests can be more sensitive to sequence variability [64] and the low level of sequence diversity observed in our data could be responsible for the absence of significance for them.

**Table 4:** Mismatch distribution analysis (parameters of demographic and spatial expansion) for phylogeographic groups of *Dalbergia miscolobium* based on chloroplast DNA (cpDNA) and nuclear ribosomal DNA (nrDNA) data.

		*Demographic expansion*		*Spatial expansion*	
*Phylogeographic groups* a	*Data*	*SSD (p-value)*	*Raggedness (p-value)*	*SSD (p-value)*	*Raggedness (p-value)*
**All populations**	cpDNA	0.016 (0.130^b^)	0.070 (0.285^b^)	0.013 (0.057^b^)	0.070 (0.150^b^)
	nrDNA	0.168 (0.000)	0.043 (0.993^b^)	0.020 (0.106^b^)	0.043 (0.188^b^)
**Central Eastern 1 (CE1)**	cpDNA	0.015 (0.051^b^)	0.160 (0.064^b^)	0.015 (0.002)	0.160 (0.072^b^)
	nrDNA	0.009 (0.056^b^)	0.110 (0.008)	0.009 (0.004)	0.110 (0.006)
**Central Eastern 2 (CE2)**	cpDNA	0.003 (0.529^b^)	0.074 (0.355^b^)	0.003 (0.358^b^)	0.074 (0.338^b^)
	nrDNA	0.007 (0.139^b^)	0.121 (0.174^b^)	0.007 (0.045)	0.121 (0.183^b^)
**Central Western 1 (CW1)**	cpDNA	0.028 (0.046)	0.280 (0.043)	0.028 (0.011)	0.280 (0.046)
	nrDNA	0.005 (0.355^b^)	0.099 (0.268^b^)	0.005 (0.215^b^)	0.099 (0.258^b^)
**Central Western 2 (CW2)**	cpDNA	0.051 (0.049)	0.137 (0.198^b^)	0.047 (0.181^b^)	0.137 (0.668^b^)
	nrDNA	0.008 (0.074^b^)	0.099 (0.037)	0.008 (0.022)	0.099 (0.037)
**Central Cerrados (CC)**	cpDNA	-	**-**	-	-
	nrDNA	0.000 (0.126^b^)	0.895 (0.912^b^)	0.000 (0.111^b^)	0.895 (0.904^b^)
**Southern Cerrados (SC)**	cpDNA	-	**-**	-	-
	nrDNA	0.459 (0.000)	0.124 (1.000^b^)	0.017 (0.593^b^)	0.124 (0.670^b^)
**Northeastern Cerrados (NC)**	cpDNA	-	-	-	-
	nrDNA	0.019 (0.109^b^)	0.072 (0.239^b^)	0.017 (0.165^b^)	0.072 (0.357^b^)
**Central Eastern (CE1+CE2)**	cpDNA	0.017 (0.028)	0.144 (0.000)	0.017 (0.000)	0.144 (0.000)
	nrDNA	0.005 (0.062^b^)	0.083 (0.029)	0.005 (0.017)	0.083 (0.020)
**Central Western (CW1+CW2)**	cpDNA	0.029 (0.025)	0.126 (0.031)	0.029 (0.000)	0.126 (0.050)
	nrDNA	0.005 (0.070^b^)	0.088 (0.020)	0.005 (0.022)	0.088 (0.023)

a The population composition of the phylogeographic groups are detailed in Table 2 and in Figure 3D.

b p-value>0.05, which means that the population set (either groups or the whole species) mismatch distribution did not differ significantly from a sudden-expansion model.

The times to the most recent common ancestor (TMRCA) for cpDNA and nrDNA haplotypes were estimated in 2.03×10^6^ (0.49–3.88) and 2.94×10^6^ (0.75–5.71) years before the present (YBP), respectively. These dates place the beginning of diversification of *D. miscolobium* lineages at the transition between Pliocene and Pleistocene, with confidence intervals spanning from Late Miocene to Middle Pleistocene. This estimated recent origin might be the cause of the low resolution of the networks. The results are very concordant between the cpDNA and nrDNA analyses, with the estimated dates being very close to each other and having overlapping confidence intervals.

## Discussion

The combined analysis of a non-coding region of the cpDNA and the ITS region from nrDNA, performed by means of four different approaches, phylogenetic relationships estimation among haplotypes, Bayesian analyses of population genetic structure, a Monmonier's algorithm to infer genetic barriers and analyses of molecular variance (AMOVAs), revealed a phylogeographic structure in *D. miscolobium* (Figure 3 and Table 2) largely concordant with some of the main phytogeographical patterns reported for the Cerrado to date. Remarkably, the observed phylogeographic structure shows a clear similarity to the Cerrado phytogeographical provinces proposed by Ratter *et al.* [11] which have been delimited based on a comprehensive sampling effort in the biome. Based on Twinspan and consensus analyses, Ratter *et al*. proposed five and four provinces respectively [11], when considering only those that are superimposed to our sampling area (Figure 3) and our data strongly support them. The main difference between Ratter's four consensus provinces and five Twinspan provinces is the status of province ‘C & SE’ (Figure 1), which is a single province in the consensus analysis but is subdivided into two in the Twinspan analysis, named 2 and 3 (Figure 3D, F) groups. In this region our genetic data also allowed the identification of two phylogeographical groups, CC and CE1+2, which correspond to groups 3 and 2, respectively in the Twinspan analysis. Therefore, the observed structure of *D. miscolobium* is in accordance to the Cerrado phytogeographical patterns observed by Ratter *et al.* [11].

Some other longstanding phytogeographical hypotheses also are corroborated by our data. Several different authors have proposed Southern Cerrados and Northeastern Cerrados as completely different units from the remaining Cerrado [10], [11], [63], [65], [66], and this pattern was also observed in our data, although a wider sampling of Northeastern Cerrados would be desirable to confirm its characteristics found by our study. In some degree, these patterns were also observed by two other phylogeographical studies [21], [22]. With regard to the separation between Eastern and Western Central Cerrados, not only the phytogeographical provinces [9]–[11] but also phylogeographic studies have showed it [20], [22]. Finally, another striking phylogeographic pattern we observed is the consistent separation into two groups of the populations in the southern limits of the Cerrado (below 20° S). They can consistently be separated into an eastern group (our SC group) and a western group (part of the CW2 group; Figure 2C, D), a pattern that have been reported by Durigan *et al.* [67] based on floristic data.

Such ‘genealogical concordances’ [17], [68] between phylogeographic and phytogeographical patterns and provinces have been found in other regions of the globe (e.g. [18], [69], [70]), but this is the first time it is explicitly shown in the Cerrado biome. Given the relatively recent diversification of *D. miscolobium*, estimated at the Pliocene/Pleistocene (see results), these ‘genealogical concordances’ suggest that a shared and persistent pattern of species diversification might have been present on the Cerrado over time. Ecological as well as historical biogeographic factors have acted collectively in shaping species diversification and distribution in the biome and the relative importance of each of these factors has been the object of considerable debate [3], [4], [71]–[73]. Castro and Martins [10] and Ratter *et al.* [11] proposed that climate (mainly duration of the dry season and mean temperature), soil fertility and drainage are the main factors responsible for the subdivisions of the Cerrado in phytogeographical provinces observed, with variation in altitude and past climatic changes also playing a part in the observed diversification. In a recent study, Simon *et al*. [73], by analyzing time-calibrated phylogenies and plant adaptations, highlighted the importance of fire and the emergence of C4 flammable grasses to the diversification of the Cerrado flora, as has also been pointed out by others [6]. For Southern Cerrados, the duration of the dry season and the contact with other vegetation types, as the Atlantic Forest, were hypothesized as probable determinant factor in differentiating eastern and western [67]. Studies based on molecular phylogenies have pointed that the diversification on the neotropical region resulted from many evolutionary forces acting in different spatial and temporal scales, which include the climatic changes of Quaternary, Neogene tectonic events and paleogeographical reorganizations [3], [12], [74]–[76]. None of these hypotheses can be discarded, and, indeed, the complex interaction among these factors across the Cerrado appears to have determined the occurrence of the biome itself [72], as well as the phytogeographical and phylogeographical patterns that occur within it. But our results suggest that some of those driving forces have persisted over time, and lead to the recent intra-specific diversification of *D. miscolobium* in a similar way to the events of speciation that are behind the current phytogeographic patterns. Future study on genetic, morphological, and physiological diversity of widespread Cerrado species might provide the basis for better understanding the contribution of each of these factors to the observed patterns of diversification.

The pattern of cpDNA diversity of *D. miscolobium* in the southern Cerrado (SC group) is also very similar to that reported for three other tree species studied in the Cerrado, *Caryocar brasiliense* [19], *Hymenaea stigonocarpa* [22] and *Plathymenia reticulata* [21]. In this region, *D. miscolobium* presents populations with low genetic diversity, probably descent from northernmost sources, a star-like network and mismatch distributions not significantly different from the demographic and expansion model distribution. Together, these patterns point to a recent range expansion [77] of the species to southern Cerrados, from northernmost sources. The retraction of the Cerrado vegetation in the present-day southern portion of Cerrado during the colder and drier periods of the LGM has been proposed by paleoenvironmental studies [14], [78] and it was one of the least probable regions of the Cerrado to persist during the LGM, according to a modeling study [79]. The subtropical SDTF [2], [80] and/or grassland species [14], [81] from the southernmost regions may have expanded northwards during the LGM, replacing the Cerrado species. Subsequent climate amelioration [15] may have allowed the range expansion of the Cerrado species southwards again. Thus, even though, among the Cerrado species, *D. miscolobium* seems to better tolerate lower temperatures, it seems to have been similarly affected by the extremely arid and cold conditions of the LGM. It is suggestive that maybe, the entirely Cerrado biota, including its more cold-tolerant species, were unable to resist to the coldest conditions of LGM times in the southern portion of the Cerrado. This same pattern was not observed for nrDNA data, whose variation, in SC group, has contrasting patterns, that is, high genetic diversity and genetic uniqueness. Rapid expansion of those populations coupled with the high rate of mutation of nrDNA, nrDNA paralogue evolution, seed/pollen bias due to cpDNA gene flow only by seeds and even hybridization/introgression are among the factors that could have been responsible for those results [29], [82]. Analyses with more markers with different evolutionary rates could help to reveal the causes of these differences between cpDNA and nrDNA diversity levels in southern Cerrado. In this regard, next-generation sequencing to produce maps of model species of Leguminosae could help to find useful markers in *D. miscolobium*.

The heterogeneous distribution of genetic diversity of *D. miscolobium* is in accordance to the commonly reported heterogeneity of Cerrado biome in terms of species distribution [3], [8], [11], [23]. Also, the high level of differentiation among populations observed in *D. miscolobium* has been reported to three other species [19], [21], [22]. Together with the widespread occurrence of exclusive haplotypes, these results reinforce the importance to treat the different Cerrado provinces as different biodiversity assemblages. Among these, two centres of genetic diversity deserve some attention: the CW2 and the CE1+CE2 regions. The high diversity of CE1+CE2 regions have been reported in the plant phylogeographic studies of *Hymenaea stigonocarpa* [22] and *Plathymenia reticulata* [21] as well as in animal [3], [83], [84] and plant [85] endemism studies. CE1+CE2 regions are also relatively close to the recently proposed principal Cerrado refugium [79]. The spatial heterogeneity and/or climate stability of this region, especially in its northern portion, could have helped to give rise to and maintain this diversity. Together with the CW1+CW2 groups, the CE1+CE2 were the only groupings of populations that did not show mismatch distributions of sequences in accordance with a recent population expansion for both cpDNA and nrDNA, in a sign that they might have been more stable than the other groups (Table 3). Based on that, we reinforce the important role that the central region of the Cerrado could have in maintaining the genetic diversity of widespread Cerrado species. With regard to CW2 group, this is the first time a phylogeographic study has performed a wide sampling in the south-western portion of the Cerrado and we have found unexpected high levels of genetic diversity and also signs of population stability on it. This region is currently under considerable economic growth and therefore, research should focus on it in order to aid conservation efforts and its sustainable exploitation.

These many patterns found rise novel questions for future consideration. Do these biogeographic patterns persist in other widespread Cerrado species? What would have occurred to species endemic to these provinces, especially those found commonly in the southern Cerrado? How did the different ecological and historical factors lead to the diversification of the Cerrado biota? Phylogeography of the Cerrado is in its infancy, and many studies are still needed to improve our understanding of the historical biogeography of this rich and threatened biome to help in its conservation and sustainable use.

## Supporting Information

Figure S1 **Results from Bayesian Analysis of Population Structure (BAPS) of** ***Dalbergia miscolobium*** **populations with a prior of k = 4. See text and** **Figure 3** **for details.**
(TIF)

Table S1 **Variable sites of aligned cpDNA sequences.**
(XLS)

Table S2 **Variable sites of aligned nrDNA sequences.**
(XLS)
