# Supplementary figures and images for: Concordance between Phylogeographical and Biogeographical Patterns in the Brazilian Cerrado: Diversification of the Endemic Tree Dalbergia miscolobium (Fabaceae)
Source: PLoS One. 2013 Dec 2;8(12):e82198. doi: 10.1371/journal.pone.0082198 (PMC3846898; doi:10.1371/journal.pone.0082198)

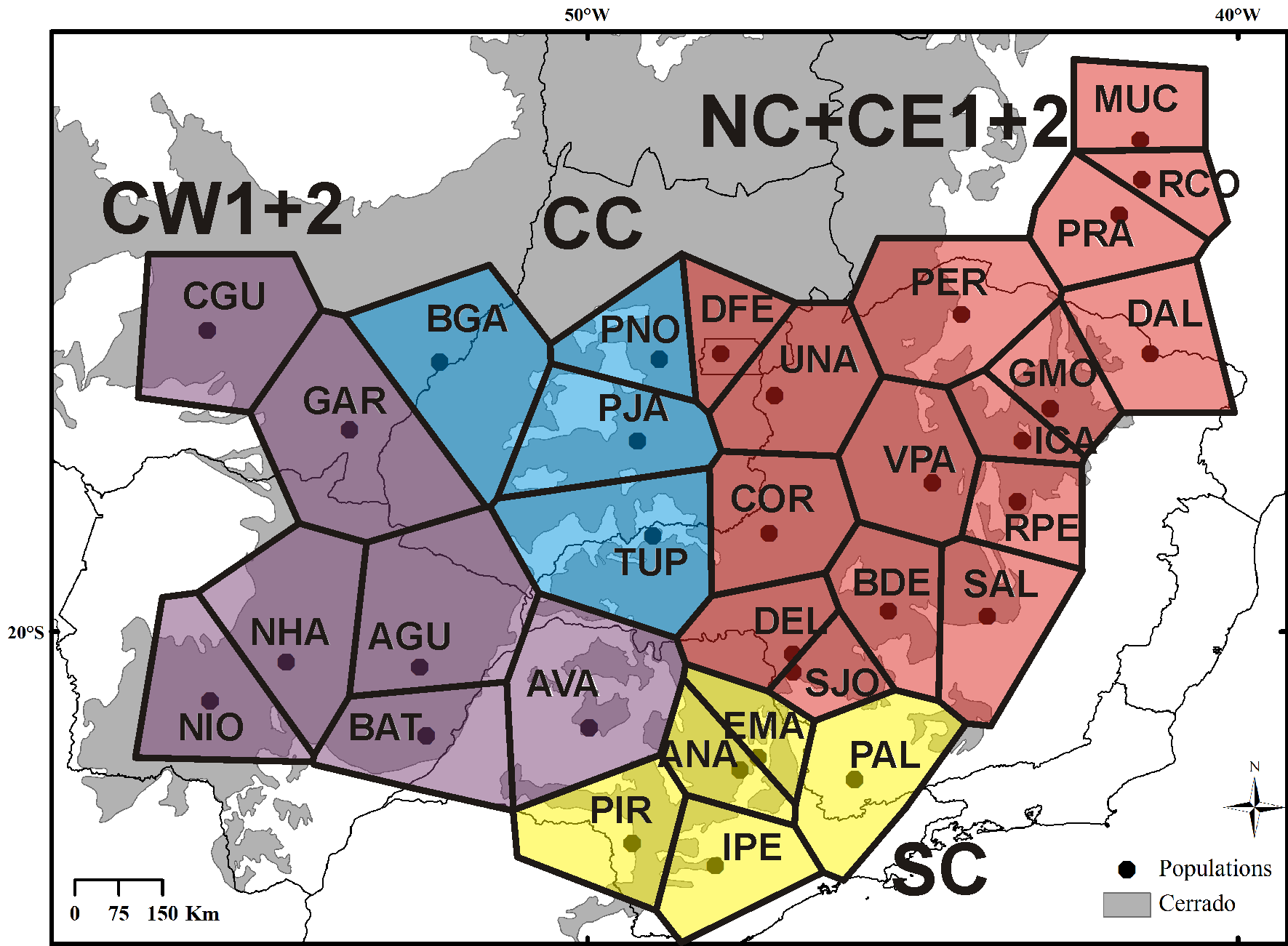

Supplement: Figure S1 — Results from Bayesian Analysis of Population Structure (BAPS) of Dalbergia miscolobium populations with a prior of k = 4. See text and Figure 3 for details. (TIF) [file pone.0082198.s001.tif]
